# Immobility in the Treatment of Diseases of Extremities1Read before the Iowa State Veterinary Medical Association, Des Moines, Iowa, September 9, 1895.

**Published:** 1895-11

**Authors:** W. L. Williams

**Affiliations:** Veterinarian to Agricultural Experiment Station, Bozeman, Montana


### IMMOBILITY IN THE TREATMENT OF DISEASES OF THE EXTREMITIES.[1]

By W. L. WILLIAMS, V.S.,
VETERINARIAN TO AGRICULTURAL EXPERIMENT STATION, BOZEMAN, MONTANA.

In the domain of therapeutics we ever recognize two great fundamental physiological factors—work and rest—and the securing or controlling one of these constitutes the chief end for which we strive.

The diseases affecting the limbs of horses constitute one of the most important groups with which the private veterinarian is called to deal, and the result is all too frequently unsatisfactory. It is a notable fact that in the treatment of these injuries veterinarians have, as a class, followed very closely the art of healing as applied to man, although it must be evident at first thought that the environments of the two are widely different, and that a measure which would suffice for the one would be inadequate or useless in the other. The principles are identical in both. In many affections of the limbs of horses the general principles of healing are readily applicable, but in the

[1] Read before the Iowa State Veterinary Medical Association, Des Moines, Iowa, September 9, 1895.

most important classes of affections we must studiously adapt art to science in order to secure the best results.

We find much difficulty confronting us in cases of sprained or ruptured ligaments or tendons, in arthritis, open joints, ostitis, wounds severing important tendons or muscles, in fractures, and in many other cases. These are all far less successfully treated in the horse than in man; not because there is any fundamental reason for it, but because in man rest can be secured without difficulty, while in the horse the securing of rest in the affected part is frequently impracticable and always involves considerable labor on the part of the veterinarian. When we remember, however, that rest from the demands of locomotion is essential in order that repair of the diseased part may take place, we should be ready to employ all possible endeavor to secure so valuable a factor in the cure of the disease.

In a measure, most lines of treatment employed in these cases unconsciously exert their chief good in producing rest. With anodyne liniments we alleviate pain, and stop thereby the dragging of the limb, while by irritants, like vesicants or cautery, we thicken the skin and parts immediately beneath in such a manner as to cause the parts to be held stiffly. These ends are brought about naturally, too, by the production of great swellings which serve to render the parts immovable. This natural production of immobility has its highest illustration in the hard, tense swellings in fractures of the limbs when let alone. It is generally supposed that fractures in man and animals must be "set" in firm splints in order for reunion to take place, but the erroneousness of this view is well demonstrated in the horses of our western plains, where in a semi-wild state they suffer all conceivable forms of fracture, yet make prompt recovery from forms which the average practitioner would count incurable because he could not apply splints. These natural methods of healing suggest forcibly that we should try harder to aid in this process.

I have noted that in a severe sprain of the hock-joint in horses the application of various medicines had no effect, the swelling remained the same or grew apace, while the pain and lameness were so intense as to bode no good result, yet all would be changed in a few hours by firmly encasing the hock in plaster of Paris. So in severe cases of ostitis, great changes are to be quickly noted when the concussion of locomotion is relieved by solid encasement. For ruptured tendons and suspensory ligaments the universal treatment seems to be cooling lotions, followed by irritants, vesicants, and cautery, but the most rational thing to do wouuld be to place the tendon at rest by removing the weight of the body from it and securing immobility of the limb. In spavin and ringbone we resort to everything else to produce anchylosis except the most rational of all, the production of immobility between the affected bones. In navicular arthritis too, especially where all bones of the foot are involved, it is quite possible that immobility would produce the desired results. In open joints the production of immobility is of quite as urgent importance as in fracture, yet it is rarely brought into use.

Surgical immobility is so rarely produced in practice that its application is not well understood and our methods are exceedingly defective. In my practice what might be termed a composite method has been employed generally, although at times I have resorted to plaster-of-Paris bandages alone. My usual plan has been to make the base of the work a well-fitted, strong shoe, well nailed on the foot of the affected member. In forging this shoe provision is made for attaching an iron brace or braces at such a point as may seem desirable, whether to a bar uniting the heels, at the toe, or at the side of the foot, almost always either at the heel or toe. The brace, usually of iron (or, better, steel), ¼ x 1 inch, is to be fitted to the shoe, but preferably not welded. It may be made to pass through a hole in the shoe and fastened underneath by means of a nut, but the screws are very weak and prone to break. The stronger and easier way is to have a slot in the shoe through which the brace can be slipped in such a manner that when brought in the position it is desired to fix it, it shall be tight and immovable, but dropped from its position it may be readily detached from the shoe. This is both strong and convenient. This metallic stay is made to extend from the shoes to a point above the part it is desired to render immobile, where it may be made to end in a variety of ways according to the part to which it is applied. Generally, if the stay is to be placed in front of the leg there is a constant tendency, when the animal moves, to cause the stay to slip to one side of the leg and eventually become loosened. This is to be prevented by broad flanges embracing the leg. If a posterior stay is used, reaching only to the upper portion of the metacarpal or metatarsal bones, no flanges are necessary, but it is usually well instead to have a slot at the top of stay through which to pass a strap to buckle around the leg. The ingenuity of the veterinarian must be constantly brought into play to adopt the stay to the special requirements of the case.

In preparing the limb for the application of the stay great care must be used to guard against galling the parts. This is reasonably well accomplished by applying cotton batting carefully with bandages, over which, at the points where friction or pressure will occur, heavy sole leather should be applied, after the edges have been thinned and the entire piece thoroughly softened in boiling water so it can be readily moulded to the parts. The leather being retained in position by means of bandages, the metal stay is now applied and over it plaster-of-Paris bandages in sufficient amount.

There are numerous methods of making and applying plaster-of-Paris bandages. A very convenient method is to take common cheesecloth, roll several yards of it in as tight a roll as possible, grasp it between strong forceps at the proper distance from the end for a bandage of convenient width, and with a sharp, strong knife cut along the jaws of the forceps.

This method gives a clean-edged bandage. The plaster-of-Paris is then spread out on a table, the bandage laid in it, and the plaster is sprinkled or thinly spread on the loose end of the bandage, the unplastered portion being gradually unrolled while that to which the plaster has been applied is rolled up in the opposite direction. The bandage should be very loosely rolled so that water can readily penetrate, and should never be long enough to be unwieldly. Generally one or two yards in length is best. The bandages are then, one at a time, dipped in a pail of hot water until saturated, the water pressed out quickly, and the bandage applied. It is generally desirable to apply the plaster from the foot up to and including the affected part, rendering the entire limb immobile up to and including the affected portion. In open joints or other wounds requiring dressing frequently the location of the wound should be carefully marked and when the plaster has hardened an opening made suitable to the needs of the case. In dressing such a wound through an opening in a plaster cast it is always to be remembered that pressure should be applied to the wound of the same degree which is applied by the cast to other parts. If this is not done the wound swells and bulges out into the opening in the cast, resulting in an unhealthy condition. In all cases but, especially in inflamed joints and other instances where there is much swelling, it will be found that within a few days the cast becomes loose owing to the diminution of the swelling. In this manner it not only loses its efficacy, but becomes a source of danger, tending to chafe the parts. In other cases where there is no appreciable swelling the immobility and pressure lead in two or three weeks to marked atrophy of the soft tissues, and doubtless, to a degree, of the bone also, making the cast fit loosely. As soon as this looseness is observed the bandage should be removed and a fresh cast applied. This is not readily done in case of fracture, owing to the danger of interference with the process of union between the bones, but in other cases it is readily accomplished.

The hock is always especially liable to chafing at the point of the os calcis, owing to the weight of the cast required to produce immobility of this joint. It is to be prevented chiefly by abundant padding, and care in supporting the plaster as far as practicable upon the stay extending from the shoe. Chafing is promptly shown by the animal constantly shifting the affected limb, and no time should be lost in removing and properly adjusting the cast once these symptoms of uneasiness are apparent.

With proper care a plaster cast may be readily retained in the treatment of various affections for ten, twelve, or more weeks. When total immobility of a member has been maintained for a number of weeks, the parts atrophy greatly and lose their power, so that when the cast or splints are removed the limb is quite unable to bear the accustomed weight, the toe turning up and the pastern touching the ground. This weakness quickly disappears without intervention and the limb soon resumes its normal strength.

The application of this method for relieving affections of the limbs should be more frequently used by veterinarians and more simple and effectual plans devised than now obtain, as this line of treatment offers at once the most humane and effectual treatment for a large proportion of the most serious and painful diseases of horses' limbs.

---

Dr. E. Wallis Hoare, of Cork, Ireland, is engaged in the production of another contribution to veterinary literature, entitled *Clinical Veterinary Medicine and Therapeutics.* This book will deal chiefly with diagnosis and treatment on the lines of a similar medical work by Dr. Burney Yeo.

